# Generation of metabolically functional hepatocyte‐like cells from dedifferentiated fat cells by Foxa2, Hnf4a and Sall1 transduction

**DOI:** 10.1111/gtc.12814

**Published:** 2020-11-10

**Authors:** Reiko Hagiwara, Yoshinao Oki, Takashi Matsumaru, Shiho Ibayashi, Koichiro Kano

**Affiliations:** ^1^ Laboratory of Cell and Tissue Biology Graduate School of Bioresource Sciences Nihon University Fujisawa Japan

**Keywords:** DFAT, direct reprogramming, gene expression analysis, hepatocyte, zonation

## Abstract

Mature adipocyte‐derived dedifferentiated fat (DFAT) cells have been identified to possess similar multipotency to mesenchymal stem cells, but a method for converting DFAT cells into hepatocytes was previously unknown. Here, using comprehensive analysis of gene expression profiles, we have extracted three transcription factors, namely Foxa2, Hnf4a and Sall1 (FHS), that can convert DFAT cells into hepatocytes. Hepatogenic induction has converted FHS‐infected DFAT cells into an epithelial‐like morphological state and promoted the expression of hepatocyte‐specific features. Furthermore, the DFAT‐derived hepatocyte‐like (D‐Hep) cells catalyzed the detoxification of several compounds. These results indicate that the transduction of DFAT cells with three genes, which were extracted by comprehensive gene expression analysis, efficiently generated D‐Hep cells with detoxification abilities similar to those of primary hepatocytes. Thus, D‐Hep cells may be useful as a new cell source for surrogate hepatocytes and may be applied to drug discovery studies, such as hepatotoxicity screening and drug metabolism tests.

## Graphical abstract

In this study, we used a comprehensive analysis of the global expression profiles of hepatocytes, mature adipocyte‐derived dedifferentiated fat (DFAT) cells, hepatic stem cells and adipocytes to identify three transcription factors—Foxa2, Hnf4a and Sall1—that can specifically induce hepatocyte differentiation in DFAT cells.

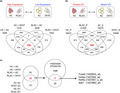

## 1 INTRODUCTION

Primary hepatocytes have been widely used for disease modeling, such as hepatitis C viral infection, and for drug metabolism and pharmacokinetics analyses (Azuma et al., 2007; Gómez‐Lechón et al., 2004; Lázaro et al., 2007). However, primary hepatocyte cultures show low proliferative capacity (Levy et al., 2015) and often exhibit progressive dedifferentiation, which often result in a relatively short lifespan and a rapid decline in liver‐specific functions (LeCluysse et al., 1996; Papeleu et al., 2003, 2006; Rogiers & Vercruysse, 1998). In order to address these issues, other cell types, such as stem cells, must be established as reliable in vitro hepatic models.

A potential alternative to primary hepatocytes is hepatocyte‐like cells derived from mesenchymal stem cells, which are capable of differentiating into most mesodermal cell types, as well as endodermal cell types (Spees et al., 2016; Wang et al., 2020). Cultured mesenchymal stem cells are of advantage in that they can be obtained in high quantities without losing their multipotency, in comparison with nonproliferative hepatocytes. These cells can differentiate into hepatocyte‐like cells that express liver‐specific genes and display the characteristic phenotypes of primary hepatocytes (Guo et al., 2017; Yin et al., 2015). Unfortunately, differentiation of these cultured stem cells is inefficient, and the resulting hepatocyte‐like cells often exhibit reduced functional capacity compared with that of primary hepatocytes.

However, direct reprogramming technology has the potential to solve these problems. Overexpression of lineage‐specific transcription factors can directly shunt terminally differentiated cells down into specific other lineages without first establishing pluripotent stem cells (iPS cells) (Shiota & Yasui, 2012). Recently, several studies have reported methods for the direct conversion of mouse fibroblasts into hepatocyte‐like cells, called induced hepatocytes (iHep cells), by the forced expression of different transcription factor combinations. Specifically, Huang et al. have shown that lentiviral vector‐mediated transduction of Gata4, Hnf1a and Foxa3, and inactivation of p19Arf could transdifferentiate mouse tail‐tip fibroblasts into iHep cells (Huang et al., 2011). Sekiya and Suzuki have also shown that Hnf4a and Foxa2 transduction in mouse embryonic fibroblasts are sufficient to direct hepatic reprogramming (Sekiya & Suzuki, 2011). Furthermore, recent studies have shown that human iHep cells can be generated from fibroblasts and adipose‐derived stem cells using a similar strategy, although implementing different factors from those used in mice (Du et al., 2014; Huang et al., 2014; Nakamori et al., 2017). These reports indicate that different combinations of master regulators of hepatocyte differentiation can be used for the direct reprogramming in various cell types. Thus, it is crucial to find the most effective combinations of factors for inducing functional hepatocytes.

To screen for master regulators of hepatocyte differentiation, candidate genes have been selected from transcription factors involved in hepatocyte differentiation during liver development (Huang et al., 2011; Sekiya & Suzuki, 2011). However, these experiments are highly time‐ and labor‐intensive, as they require repeated transfections of candidate genes into cells using viral vectors and screening for induction of hepatocyte differentiation. Recently, several studies have used comprehensive gene expression analysis to show novel gene signatures associated with liver diseases (Smalling et al., 2013) and to further identify novel genes that are possibly associated with heart diseases (Li et al., 2019). Thus, comprehensive gene expression analysis may be a useful tool for selecting candidate genes and identifying efficient master regulators of hepatocyte differentiation.

In mammals, terminally differentiated cells have been determined to be generally incapable of reversing the differentiation process (Brockes & Kumar, 2005; Carlson, 2005). However, we have reported the use of in vitro dedifferentiation strategy, called the ceiling culture method, which exploits the buoyant properties of mature adipocytes. Using this technique, the phenotype of fully differentiated adipocytes could be switched to a more primitive one, featuring an extensive proliferative ability (Yagi et al., 2004). This newly established cell line, called dedifferentiated fat (DFAT) cells, then exhibits characteristics comparable to those of adipose‐derived stem cells (ASCs), found in the stromal vascular fraction of adipose tissue (Matsumoto et al., 2008). DFAT cells exhibit adipogenic, osteogenic, chondrogenic, cardiomyogenic, angiogenic, myogenic and neurogenic potentials under proper conditions both in vitro and in vivo (Kazama et al., 2008; Matsumoto et al., 2008; Nobusue et al., 2008; Oki et al., 2008; Yamada et al., 2014). Thus, compared to ASCs, DFAT cells have unique advantages in terms of their abundance, ease of isolation and homogeneity. These properties make DFAT cells applicable to drug discovery studies and cell‐based therapies for organ failure. However, it is not yet clear whether mature adipocyte‐derived DFAT cells are able to give rise to functional hepatocytes.

Thus, in this present study, we investigated whether functional hepatocytes can be generated from mature adipocyte‐derived DFAT cells through a direct reprogramming method. Through comprehensive analysis of the global expression profile of hepatocytes, adipocytes, hepatic stem cells and DFAT cells, we identified three transcription factors—Foxa2, Hnf4a and Sall1 (FHS)—that can induce hepatocyte differentiation. After hepatogenic induction, FHS‐infected DFAT cells showed typical epithelial morphology, similar to primary hepatocytes, and further exhibited increased expression and function of hepatocyte‐specific genes. Additionally, metabolism of hepatotoxic drugs resulted in injury to hepatocytes derived from DFAT (D‐Hep) cells. These findings showed that D‐Hep cells exhibit functional features of mature hepatocytes in vitro, including detoxification capabilities.

## 2 RESULTS

### 2.1 Extraction and identification of master regulators of hepatocyte differentiation in DFAT cells by comprehensive gene expression analysis

To identify master regulators of hepatocyte differentiation to induce conversion of DFAT cells into hepatocytes, we analyzed the gene expression profiles of mature adipocytes (AC), DFAT cells, hepatocytes (HC: as the target cell) and resident liver stem cells (RLSC: as hepatic stem cells) by using the Affymetrix GeneChip Mouse Genome Array, which can detect the regulation of 45,101 probe sets. The microarray data discussed in this publication were previously deposited in NCBI’s GEO database (Barrett et al., 2009), which is accessible through GEO accession numbers GSM4732619, GSM4732620, GSM4732621, GSM4732622, GSM4732623, GSM4732624 (series GSE156495), GSM785818, GSM785819, GSM785820 and GSM162863.

**FIGURE 1:**
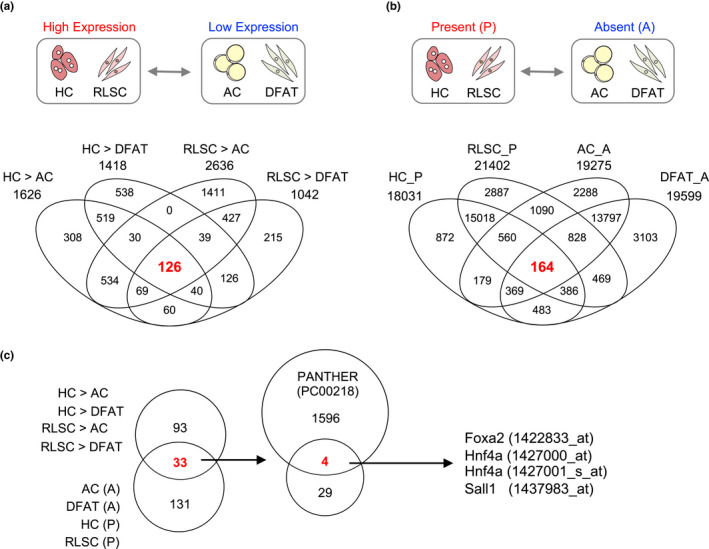
Identification of candidate master regulators of hepatocyte differentiation. (a) Fold change. Venn diagram shows the overlap of common probe sets. The diagram labels indicate the four comparison pairs. HC > AC, up‐regulated (>log_2_2) in HC compared to AC; RLSC > AC, up‐regulated (>log_2_2) in RLSC compared to AC; HC > DFAT, up‐regulated (>log_2_2) in HC compared to DFAT; and RLSC > DFAT, up‐regulated (>log_2_2) in RLSC compared to DFAT. (b) MAS5.0 call. Venn diagram shows the overlap of common probe sets. HC_P, probe sets present in HC; RLSC_P, probe sets present in RLSC; AC_A, probe sets absent in AC; and DFAT_A, probe sets absent in DFAT. (c) Analysis of overlapping genes. Venn diagram shows the overlap of common probe sets in the (a) vs. (b) (left), and probe sets for transcription factors (right). Annotation information for "transcription factor" (Class ID: PC00218) was obtained from the PANTHER Classification System

By comparing the microarray data obtained from DFAT cells and HCs, we expected to identify master regulators of hepatocyte differentiation that were initially absent in DFAT cells. However, we included data from RLSCs to narrow down the candidates to only those genes required for the commitment to a hepatogenic cell fate, as HCs express a number of genes related to mature hepatic functions. Furthermore, DFAT cells, which have the ability to redifferentiate into adipocytes, can also express differentiation regulators that are common to both adipocytes and hepatocytes. Thus, to remove those common regulators, we additionally analyzed ACs. First, we determined the number of highly expressed probe sets in HCs and RLSCs to extract genes specific for hepatocyte differentiation. Probe sets with ≥fourfold increased expression were considered to have expression differences. Compared with ACs, 1626 differential probe sets were up‐regulated in HCs, whereas 2,636 differential probe sets were up‐regulated in RLSCs. In contrast, compared with DFATs, 1,418 differential probe sets were up‐regulated in HCs, whereas 1,042 differential probe sets were up‐regulated in RLSCs. In all four comparison groups (HC > AC, RLSC > AC, HC > DFAT and RLSC > DFAT), 126 differential probe sets were identified in common (Figure 1a).

Next, to provide an accurate count of the number of probes that were expressed in HCs and RLSCs, an assessment of the detection sensitivity was needed. If signal values fall below a reliable detection limit, the values are deemed not useful for determining signal ratios. To determine which genes showed a reliable level of RNA expression, the MAS5.0 call was used in analyzing the signals based on absent/present calls for each probe set. First, those genes that showed reliable signal levels in biological replicates were identified. When a transcript was reliably detected, it was given a detection call of “P” (present), and when it was not detected, it was given a detection call of “A” (absent). In total, 18,031 differential probe sets had P‐call values in HCs, 21,402 differential probe sets had P‐call values in RLSCs, 19,275 differential probe sets had A‐call values in ACs, and 19,599 differential probe sets had A‐call values in DFAT cells. In all 4 comparison groups (HC_P, RLSC_P, DFAT_A and AC_A), 164 differential probe sets were identified in common (Figure 1b).

We then asked which of these probe sets were common among the two comparative analyses. This has yielded a total of 33 differential probe sets. Moreover, transcription factors were extracted using the transcription factor annotation in the PANTHER Classification System database (class ID PC00218) because the differentiation determinant factors were expected to be transcription factors. We then identified Foxa2, Hnf4a and Sall1 as master regulators of hepatocyte differentiation in DFAT cells (although Hnf4a genes were represented by more than one probe set on the microarray) (Figure 1c). Thus, we successfully extracted Foxa2, Hnf4a and Sall1 as hepatocyte differentiation regulators in DFAT cells.

### 2.2 Expression of hepatocyte‐specific markers and function after hepatic induction of DFAT cells transfected with Foxa2 (F), Hnf4a (H) and Sall1 (S)

**FIGURE 2:**
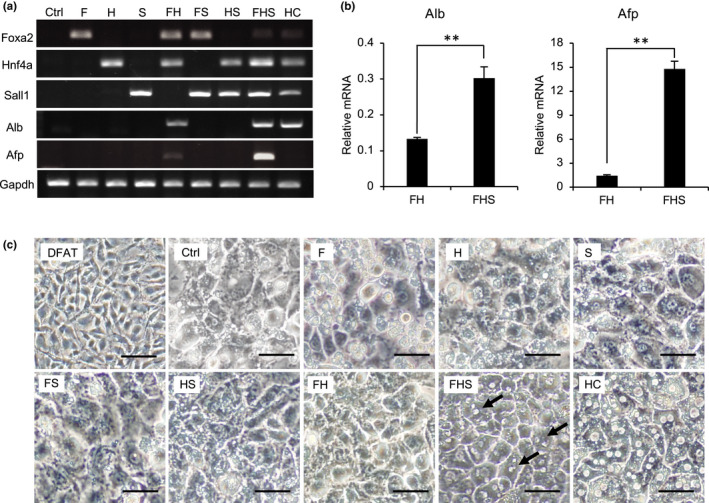
Effect of combined expression of Foxa2, Hnf4a and Sall1 on hepatic differentiation of DFAT cells. (a) DFAT cells transfected with 3 genes (Foxa2 (F), Hnf4a (H) and Sall1 (S)) or infected with the corresponding GFP vector (Ctrl) were exposed to induction medium of differentiation for 14 days. The expression levels of Foxa2, Hnf4a, Sall1 and liver genes (Alb and Afp) were measured using RT‐PCR. Gapdh was used as an internal control. (b) Real‐time PCR analyses showed the presence of hepatic marker genes for Alb and Afp in FH‐ and FHS‐expressing DFAT cells after hepatic differentiation. Mean values ± *SEM*. were then normalized to Gapdh and expressed relative to normal hepatocytes from adult mice. *p*‐Value was calculated using Student's *t* test (**p < .01). (c) Phase‐contrast microscopy showing DFAT cells and morphological changes of the cells after hepatic induction. Arrows show binucleated FHS‐DFAT cells. Scale bars represent 50 μm. All quantitative data are mean ± *SD*. (*n* = 3 experiments)

To determine whether the extracted transcription factor genes (referred to as FHS) could induce DFAT cells to differentiate into hepatocytes, DFAT cells were transduced with a mixture of viruses expressing each factor alone, in combination, or with a GFP retrovirus (negative control). Primary HCs were used as controls for hepatic differentiation. DFAT cells expressing the FHS factors as three factors alone or in combination were then replated onto collagen‐coated dishes and cultured for an additional 14 days. We confirmed the expression of the transfected genes in these DFAT cells. Gene expression of albumin (Alb) and alpha‐fetoprotein (Afp), often used as specific indicators of hepatic maturity, was observed in FH‐ and FHS‐DFAT cells, whereas no expression of these hepatic markers was detected in DFAT cells infected with a combination of FS, HS or each factor alone. Furthermore, FHS‐DFAT cells exhibited Foxa2, Hnf4a and Sall1 expression levels similar to those detected endogenously in HCs (Figure 2a). Real‐time PCR analysis showed that Alb and Afp expression was strongly induced in FHS‐DFAT cells compared to FH‐DFAT cells (Figure 2b). After 14 days of hepatic differentiation induction, FHS‐DFAT cells were converted from a fibroblast‐like morphology to an epithelial cell‐like shape, similar to primary HCs (Figure 2c). Furthermore, in hepatic differentiation‐induced FHS‐DFAT cells, binucleated cells similar to primary HCs were observed to appear (Figure 2c, arrows). These results indicate that the combination of the three transcription factors, Foxa2, Hnf4a and Sall1, is required to effectively induce DFAT cells into hepatocytes in vitro. In combination, the three transcription factors induced DFAT cells to express hepatic genes, indicating the potential ability to induce hepatic conversion of DFAT cells in vitro. Hence, we referred to these cells as D‐Hep (DFAT cell‐derived hepatocyte‐like) cells.

**FIGURE 3:**
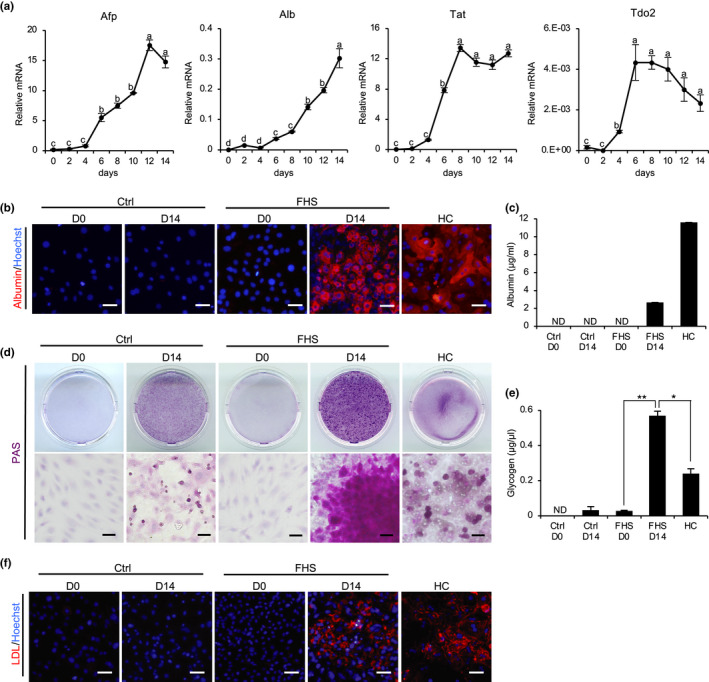
Hepatic functions in FHS‐DFAT cells before and after hepatic induction. (a) FHS‐DFAT cells were cultured in hepatic medium for 14 days. The temporal expressions of hepatocyte marker genes Afp, Alb, Tat and Tdo2 were examined by real‐time PCR analyses in FHS‐DFAT cells during hepatic differentiation. Mean values ± *SEM*. were normalized to Gapdh and expressed relative to normal hepatocytes from adult mice. *p*‐Value was calculated using Tukey's honestly significant difference test (a–d: *p* < .01). (b) FHS‐DFAT cells and GFP‐DFAT cells (Ctrl) before (D0) and after (D14) induction of differentiation and HCs were subjected to immunostaining with anti‐Alb (red) antibodies. Nuclei were stained with Hoechst 33342 (blue). (c) The Alb secretion capacity was examined by ELISA. ND, not detected. (d) Glycogen stores were showed by PAS staining. (e) Total cellular glycogen of FHS‐DFAT cells and HCs was examined. Superscript letters indicate significant (*p* < .01) differences. *p*‐Value was calculated using Tukey's honestly significant difference test. ND, not detected. (f) FHS‐DFAT cells and HCs were cultured in a medium containing LDL (red) for 4 hr, and immunohistochemistry was carried out. Nuclei were counterstained with Hoechst 33342 (blue). All scale bars represent 50 μm. All quantitative data are means ± *SD*. (*n* = 3 experiments)

As D‐Hep cells exhibited specific characteristics of hepatocytes (Figure 2a–c), we wanted to determine whether they could carry out unique functions of mature hepatic cells. To do this, we examined gene expression levels of markers in cells cultured in the induction medium during a time course, with assessments every 2 days. After 14 days of hepatic differentiation induction, we observed increased expression levels of Afp, a fetal‐specific hepatic marker; Alb; tyrosine aminotransferase (Tat); and tryptophan 2,3‐dioxygenase (Tdo2), a hepatic marker (Figure 3a). The expression level of Alb in D‐Hep cells was further evaluated using immunochemical staining. After 14 days of hepatic differentiation induction, D‐Hep cells were found to be positive for Alb, similar to primary HCs (Figure 3b). In addition, the Alb levels in the supernatants of D‐Hep cells were significantly higher than those of the control groups (Figure 3c), indicating the ability of D‐Hep cells to produce Alb. Moreover, D‐Hep cells displayed numerous hallmark features of mature hepatocytes, including glycogen storage (Figure 3d, e) and intake of low‐density lipoprotein (LDL) (Figure 3f). These results indicate that D‐Hep cells acquire the functions of mature hepatocyte‐like cells by inducing hepatic differentiation.

### 2.3 D‐Hep cells exhibit functional characteristics of Zone 3 hepatocytes

Functional mature hepatocytes located in the periportal and perivenous zones (proposed as Zones 1 and 3, respectively) of the liver lobule, although histologically indistinguishable, exhibit remarkable differences in their levels and activities of various enzymes and other proteins (Jungermann & Katz, 1989). Here, we examined whether D‐Hep cells possessed the unique functions of Zone 1 or Zone 3 hepatocytes.

**FIGURE 4:**
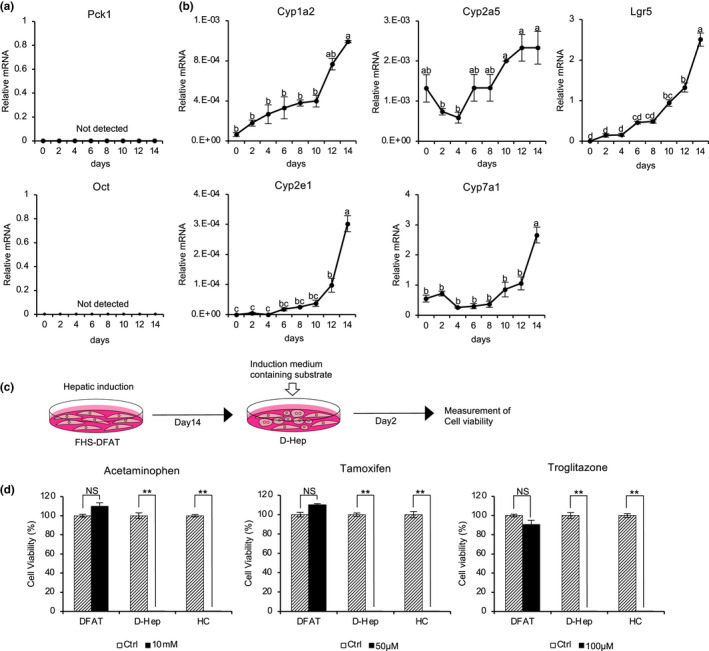
Expression of Zone 1 and Zone 3 hepatocyte markers and function in D‐Hep cells. (a) FHS‐DFAT cells were cultured in hepatic medium for 14 days. The temporal expression of Zone 1 hepatocyte marker genes (Oct and Pck1) was examined by real‐time PCR analysis in D‐Hep cells during hepatic differentiation. (b) The temporal expression of Zone 3 hepatocyte marker genes (Cyp2a5, Cyp1a2, Cyp2e1, Cyp7a1 and Lgr5) was examined using real‐time PCR analysis in D‐Hep cells during hepatic differentiation. Mean values ± *SEM*. were normalized to Gapdh and expressed relative to normal hepatocytes from adult mice. Superscript letters indicate significant (*p* < .01) differences. *p*‐Value was calculated using Tukey's honestly significant difference test. (c) Schematic diagram of the experimental procedure. (d) The cell viabilities of DFAT cells (blue), D‐Hep cells (red) and primary hepatocytes (black) were assessed by ATP assay after a 48‐hr exposure to the three compounds (acetaminophen, tamoxifen and troglitazone). *p*‐Value was calculated using Student's *t* test (**p < .01). NS, not significant. All scale bars represent 50 μm. All quantitative data are means ± *SD*. (*n* = 3 experiments)

We first assessed the expression of ornithine transcarbamylase (Oct) and phosphoenolpyruvate carboxykinase 1 (Pck1), which are specifically expressed in Zone 1 hepatocytes, and found that neither were detectable during the culture period after the induction of hepatic differentiation (Figure 4a). Next, we examined the expression of cytochrome P450 (CYP) genes, such as Cyp1a2, Cyp2a5, Cyp2e1, Cyp7a1 and leucine‐rich repeat‐containing G protein‐coupled receptor 5 (Lgr5), which are specifically expressed in Zone 3 hepatocytes. The expression of Cyp1a2 and Cyp2e1 genes has been observed to increase rapidly after 10 days of hepatic differentiation induction and showed the highest values at 14 days. The expression of Cyp2a5 and Lgr5 genes increased rapidly after 10 and 12 days of hepatic differentiation induction, respectively (Figure 4b). Thus, key CYP genes, including Cyp1a2, Cyp2a5, Cyp2e1 and Cyp7a1, were found to be remarkably up‐regulated in D‐Hep cells after inducing hepatic differentiation. Considering that detoxification is a unique characteristic of Zone 3 hepatocytes, we evaluated the detoxification abilities of D‐Hep cells. D‐Hep cells were incubated with three substrates, namely acetaminophen, tamoxifen and troglitazone, which are then acted on by CYP enzymes, generating toxic metabolites. Then, the cell viability was measured (Figure 4c). None of the substrates affected the viability of FHS‐DFAT cells when they were cultured in a medium supplemented with those compounds. In contrast, the viability of D‐Hep cells was markedly reduced to levels similar to those detected in primary HCs for each of the three test compounds, with most of the cells dying (Figure 4d). Taken together, these results indicate that D‐Hep cells exhibit functional characteristics of Zone 3 hepatocytes.

**FIGURE 5:**
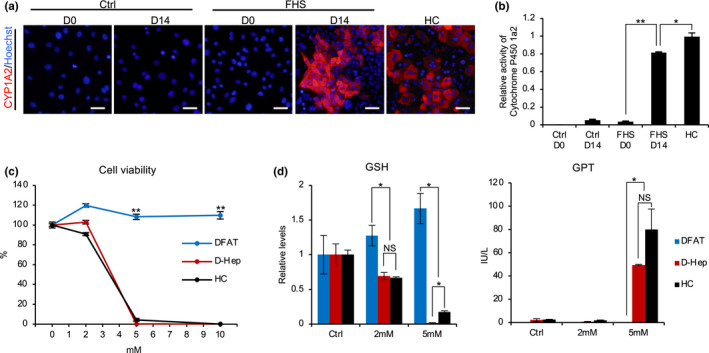
Cyp1a2‐mediated drug metabolism in D‐Hep cells. (a) FHS‐DFAT cells and GFP‐DFAT cells (Ctrl) before (D0) and after (D14) induction of differentiation and primary hepatocytes (HC) were subjected to immunostaining with anti‐Cyp1a2 (red) antibodies. Nuclei were stained with Hoechst 33342 (blue). (b) Cyp1a2 activity in FHS‐DFAT cells and HCs was measured. *p*‐Value was calculated using Student's *t* test (*p < .05, **p < .01). (c) The cell viabilities of DFAT cells (blue), D‐Hep cells (red) and HCs (black) were assessed by ATP assay after a 48‐hr exposure to different concentrations of APAP. *p*‐Value was calculated using Tukey's honestly significant difference test compared to D‐Hep cells. (**p < .01). (d) GSH content and GPT activity in cells treated as in C. *p*‐Value was calculated using Tukey's honestly significant difference test (*p < .05). NS, not significant. Scale bars represent 50 μm. All quantitative data are means ± *SD*. (*n* = 3 experiments)

Acetaminophen (APAP) produces toxic reactive metabolites by Cyp1a2. Excess production of this metabolite can lead to the depletion of hepatic glutathione, which in turn result in hepatocyte cell death (Ramachandran & Jaeschke, 2018). Thus, we investigated whether Cyp1a2‐mediated drug metabolism of APAP, which is a characteristic of Zone 3 hepatocytes, occurs in D‐Hep cells. Using immunocytochemical staining, we observed significant increases in Cyp1a2‐positive cells in D‐Hep cultures at 14 days after the induction of hepatic differentiation (Figure 5a). Moreover, the enzymatic activity of Cyp1a2 was determined to be significantly up‐regulated after hepatic differentiation, compared to that before the induction of differentiation (Figure 5b). We then exposed D‐Hep cells to different concentrations of APAP and evaluated the cell viability by measuring intracellular ATP levels. Strikingly, we observed a loss of viability in D‐Hep cells at 5 and 10 mM APAP treatments (Figure 5c). The levels of intracellular glutathione (GSH) in D‐Hep cells 48 hr after the addition of APAP were determined to be significantly lower in both the 2 and 5 mM APAP groups than in the control group. In particular, intracellular GSH in D‐Hep cells treated with 5 mM APAP was depleted (Figure 5d). Glutamic pyruvic transaminase (GPT) is released into the blood when hepatocytes are damaged by APAP and increased in supernatants following cultured hepatocyte damage. The GPT concentration in the culture supernatant of D‐Hep cells 48 hr after the addition of APAP was significantly higher in both 5 mM APAP and the primary HC groups than in the control or 2 mM APAP groups (Figure 5d). These results suggested that D‐Hep cells possess the ability to detoxify APAP, similar to primary HCs. Taken together, these results suggest that D‐Hep cells may possess the detoxification abilities of Zone 3 hepatocytes.

## 3 DISCUSSION

In previous studies, candidate genes for hepatic fate‐inducing factors have been selected from transcription factors that are reported to play a role in liver development and hepatocyte function (Willenbring, 2011). Screening for candidate factors that efficiently induce direct reprogramming has historically been carried out by infecting mouse or human fibroblasts with a viral vector that combines all candidate factors selected based on previous reports (Du et al., 2014; Huang et al., 2011; Sekiya & Suzuki, 2011). These conventional screening methods are considered to be time‐consuming and costly, but not always successful. Thus, in this present study, we attempted to identify the transcription factors that induce functional hepatocyte differentiation from DFAT cells by comprehensively analyzing gene expression profiles. We hypothesized that transcription factors that are only expressed in both hepatic stem cells and hepatocytes, but not in DFAT cells and adipocytes, could give DFAT cells the ability to differentiate into functional hepatocytes. We thus carried out comparative analyses using not only the gene expression profiles of hepatic stem cells and hepatocytes, but also the expression profiles of DFAT cells and adipocytes. Using this approach, we have successfully extracted three factors, namely Foxa2, Hnf4a and Sall1, which have been identified to have the ability to convert DFAT cells into functional hepatocytes (Figures 1, 2, 3). Interestingly, of the three factors identified in this study, Foxa2 and Hnf4a were consistent with previous reports that identified liver transcription factors by conventional screening methods (Huang et al., 2011; Sekiya & Suzuki, 2011). These results indicate that comprehensive analysis using gene expression profiles can extract transcription factors that induce DFAT cells into hepatocytes. We suggest that comprehensive gene expression analysis might become a useful tool for efficient and accurate identification of direct reprogramming factors.

In this study, we extracted not only the hepatocyte master regulators Foxa2 and Hnf4a, but also Sall1, by using our comprehensive gene expression analysis approach (Figure 1a–c). DFAT cells induced with Foxa2, Hnf4a and Sall1 exhibited epithelial cell‐like morphology with binuclear similar to primary HCs and strongly expressed Alb and Afp, compared with cells induced only with Foxa2 and Hnf4a (Figure 2a–c). These results suggest that Sall1, in combination with Foxa2 and Hnf4a, also plays a crucial role in hepatocyte differentiation. However, a functional role for Sall1 in direct reprogramming, hepatic differentiation and liver development has not yet been reported. Sall1 has been identified as a multi‐zinc finger transcription factor that is expressed during embryogenesis in the central nervous system, limb buds, heart and kidneys (Karantzali et al., 2011; Nishinakamura et al., 2001). It is also highly expressed in ES cells, and Karantzali et al. showed a novel role for Sall1 as a member of the transcriptional network that regulates stem cell pluripotency (Karantzali et al., 2011). Overexpression of Sall1 during embryoid body formation suppressed differentiation into mesoderm and ectoderm, but it did not affect differentiation into endoderm (Karantzali et al., 2011). In fact, Sall1 affects the induction of neuronal and cardiogenic differentiation markers in ES cells. It likely acts as a transcriptional repressor by localizing to heterochromatin and interacting with components of the nucleosome remodeling and deacetylase complex (Lauberth & Rauchman, 2006). Based on these findings, we suggest that Sall1 acts as a transcriptional repressor that controls differentiation into mesoderm and ectoderm, thereby mediating the conversion of FHS‐DFAT cells into hepatocytes. It is conceivable that Sall1 promotes the action of Foxa2 and Hnf4a, which can strongly induce direct hepatic reprogramming.

The mechanism by which FHS‐infected DFAT cells generate Zone 3 hepatocytes remains unclear. Liver zonation is governed by canonical Wnt/β‐catenin signaling. Its signaling activity is highest around the central vein, and nuclear β‐catenin directly regulates the activation of the expression of drug‐metabolizing enzymes, such as hepatic CYP genes (Benhamouche et al., 2006; Burke et al., 2009; Sekine et al., 2006). Wnt/β‐catenin signaling is activated by R‐spongin that exerts its function by binding to Lgr5, a receptor that is highly expressed in Zone 3 hepatocytes (Braeuning et al., 2006; Clevers et al., 2014; Yoon & Lee, 2012). Additionally, Lgr5 gene expression in FHS‐DFAT cells increased rapidly 10 and 12 days after inducing hepatic differentiation (Figure 4b). It has been reported that CHIR99021 (CHIR), a highly selective inhibitor of glycogen synthase kinase 3β (GSK3β), is a potent inducer that activates Wnt/β‐catenin signaling (Chen et al., 2014). Consistent with this result, Sall1, a master regulator of hepatocyte differentiation from DFAT cells in this study, was found to cooperatively promote Wnt signaling with β‐catenin (Sato et al., 2004). Taken together, these results suggested that FHS‐DFAT cells differentiated into Zone 3 hepatocytes as a result of Lgr5 expression from the early stages of differentiation (Figure 4b), with R‐spondin1 and CHIR99021 contained in the hepatic medium. Thus, the zone specificity of D‐Hep cells could be directed to Zone 1 hepatocytes by inhibition of Wnt/β‐catenin signaling.

Studies of D‐Hep cells may provide a novel approach for examining hepatocyte differentiation and liver diseases. More detailed drug metabolism experiments and hepatotoxicity evaluation tests must also be carried out to examine whether D‐Hep cells can be used for drug discovery research. In addition, in order to evaluate the possible use of D‐Hep cells for regenerative medicine, we would like to determine whether the transplantation of these cells has a therapeutic effect by using mice with liver injury. Although there are still many questions left unanswered, we believe that DFAT cells may be applicable to hepatotoxicity screening and drug metabolism tests.

## 4 EXPERIMENTAL PROCEDURES

### 4.1 GeneChip microarray hybridization and data acquisition

Primary mouse ACs were isolated using the methods described by Nobusue and Kano (Nobusue et al., 2008). The mouse preadipocyte cell line DFAT was established from dedifferentiated mature adipocytes of GFP transgenic mice by ceiling culture (a method to culture lipid‐containing adipocytes based on their lipid buoyancy) (Nobusue et al., 2008). Total RNA was extracted from differentiated and dedifferentiated mouse ACs using TRIzol reagent (Invitrogen) and RNeasy Mini Kit (Qiagen), according to the manufacturer's instructions. The quality of RNA was assessed using an Agilent 2100 Bioanalyzer (Agilent Technologies). Then, the RNA samples from AC and DFAT cells were labeled using the GeneChip One‐Cycle Target Labeling and Control Reagent package (Affymetrix) and then hybridized to the Affymetrix GeneChip Mouse Genome 430 2.0 Array, according to the manufacturer's instruction. Fluorescent images were then visualized using a GeneChip Scanner 3000 (Affymetrix). Expression data and raw expression data (CEL files) were generated using the GeneChip Operating System software (Affymetrix). The raw and processed microarray data were deposited in the Gene Expression Omnibus (GEO, https://www.ncbi.nlm.nih.gov/geo/) of the National Center for Biotechnology Information (NCBI), which is a public and freely available platform, and are accessible through the GEO Series accession number GSE156495 (https://www.ncbi.nlm.nih.gov/geo/query/acc.cgi?acc=GSE156495).

### 4.2 Microarray analysis

A flow chart displaying the major steps involved in the microarray analysis has been provided in Figure S1. Gene expression profiling was carried out on total RNA samples from ACs, DFAT cells, HCs and RLSCs. HC and RLSC microarray datasets (GSM785818, GSM785819, GSM785820 and GSM162863) were then obtained from NCBI’s GEO. Microarray analyses were initially carried out in R (http://www.r‐project.org/), using the Bioconductor package affy (http://www.bioconductor.org/packages/2.0/bioc/html/affy.html).

The raw data were normalized using the RMA (robust multiarray average).

Absolute value was taken by using the mas5calls function. The Spotfire DecisionSite for Functional Genomics software package (TIBCO Software) was used for the fold change analysis (>log_2_2). Transcription factors were then identified from the PANTHER Classification System database (Thomas et al., 2003). Using the PANTHER Protein Class Ontology browser, we were able to retrieve genes classified as “Transcription Factor” (PC00218) in mus musculus. Common genes were selected by fold change analysis and absolute value and transcription factors. These genes were used as candidate master regulators of hepatocyte differentiation.

### 4.3 Cell culture

DFAT cells were cultured under a humidified atmosphere of 5% CO_2_ and 95% air at 37°C on collagen type I‐C (Nitta Gelatin)‐coated tissue 40‐mm culture dishes (TPP) containing Dulbecco's modified Eagle's medium (DMEM) (Nissui Pharmaceutical) supplemented with 10% fetal bovine serum (FBS) (Moregate BioTech). DFAT cells were grown to semiconfluence for differentiation. Hepatic differentiation was induced by changing the medium to SHM + YAC (Chen et al., 2012), namely DMEM/F12 (Gibco) containing 2.4 g/L NaHCO_3_ and l‐glutamine, which was supplemented with 5 mM HEPES (Sigma‐Aldrich), 30 mg/L l‐proline (Sigma‐Aldrich), 0.05% BSA (Sigma‐Aldrich), 10 ng/ml epidermal growth factor (PeproTech), insulin–transferrin–serine (ITS)‐X (Gibco), 10^–7^ M dexamethasone (Dex)(Sigma‐Aldrich), 10 mM nicotinamide (Wako), 1 mM ascorbic acid‐2 phosphate (Sigma‐Aldrich), 10 mM Y‐27632 (Wako), 0.5 mMA83‐01 (Wako) and 3 mM CHIR99021 (Axon Medchem). After 2 days, the culture medium was replaced with SHM + YAC, which was supplemented with 20 ng/ml oncostatin *M* (OsM) (Wako), 10^−6^ M Dex and 500 ng/ml R‐spondin1 (PeproTech). The cells were then cultured for 12 days, with fresh medium provided every 2 days.

### 4.4 Primary hepatocyte isolation and culture

Primary hepatocytes were isolated from adult mouse livers by standard two‐step collagenase digestion. Briefly, the liver was preperfused through the portal vein with calcium‐ and magnesium‐free Hank's/EGTA solution and then perfused with approximately 40 ml of Hank's solution containing 0.05% collagenase (Sigma‐Aldrich). All perfusion solutions were preheated at 37°C. The liver was removed and was later placed in a dish with Hank's solution and agitated gently after opening the liver capsule. The suspension of released cells was filtered through a 100‐µm nylon mesh to remove cell clumps. The cell suspension was then transferred to 50‐ml tubes, which were filled with Hank's solution, and the cells were collected via centrifugation at 50 *g* for 7 min. Then, the cells were resuspended in Percoll buffer (90% Percoll (GE Healthcare), 1 × Hank's), and dead cells were removed via centrifugation at 50 *g* for 15 min. The cells were washed in 40 ml/tube of Hank's solution twice via centrifugation at 50 *g* for 2 min. The purified hepatocytes were used for various experiments. Isolated cells were seeded at 2.2 × 10^4^ cells/cm^2^ in SHM containing 10% FBS onto collagen type I‐C (Nitta Gelatin)‐coated 40‐mm tissue culture dishes (TPP).

### 4.5 RT‐PCR analysis

Total RNA was isolated from cells using TRIzol reagent (Invitrogen), according to the manufacturer's instructions. Portions (1 mg) of the DNase I‐treated RNA were subjected to reverse transcription (RT) with the use of SuperScript™ IV VILO™ Master Mix (Applied Biosystems). The reverse transcripts were used as templates for analysis of gene expression levels using a PX2 thermal cycler and TaKaRa Ex Taq (Takara Bio), according to the manufacturer's instructions. The following primer sets were used for hepatic markers: Foxa2 (forward 5'‐GGAGCCGTGAAGATGGAA‐3', reverse 5'‐CGCCCACACATAGGATGACA G‐3'); Hnf4a (forward 5'‐GCGACTCTCTAAAACCCTTGC‐3’, reverse 5'‐TTCTTCCTCACGCTCCTCCT‐3'); Sall1 (forward 5'‐CACAAGAAACCCAAGTGGCG‐3', reverse 5'‐GGACCACTGCGTTTGTGAAC‐3'); Alb (forward 5'‐CACCTTTCCTATCAACCCCACTA‐3', reverse 5'‐AGCAGTCAGCCAGTTCACCA‐3'); and Afp (forward 5'‐GGACTGCTCGAAACATCCCA‐3', reverse 5'‐TCTCGGCAGGTTCTGGAAAC‐3'). Primers for d‐glyceraldehyde‐3‐phosphate dehydrogenase (Gapdh) were used as an internal standard marker (forward 5'‐GGGAAGCTTGTCATCAATGG‐3', reverse 5'‐GTTGTCATGGATGACCTTGG‐3'). PCR conditions were as follows: 5 min at 94°C followed by 30 cycles of 30 s at 94°C, 30 s at 60°C and 1 min at 72°C, with a final elongation step of 5 min at 72°C. Aliquots of PCR product were analyzed on a 2% agarose gel containing Atlas ClearSight (Bioatlas).

### 4.6 Real‐time PCR analysis

The probes for Alb (GenBank accession no. NM_009654.3; Mm00802090_m1), Afp (GenBank accession no. NM_007423.4; Mm00431715_m1), Tat (GenBank accession no. NM_146214.3; Mm01244282_m1), Tdo2 (GenBank accession no. NM_019911.2; Mm00451269_m1), Cyp1a2 (GenBank accession no. NM_009993.3; Mm00487224_m1), Cyp2e1 (GenBank accession no. NM_021282.2; Mm00491127_m1), Cyp2a5 (GenBank accession no. NM_007812.4; Mm00487248_g1), Cyp7a1 (GenBank accession no. NM_007824.2; Mm00484150_m1), Lgr5 (GenBank accession no. NM_010195.2; Mm00438890_m1), Otc (GenBank accession no. NM_008769.3; Mm00493267_m1) and Pck1 (GenBank accession no. NM_011044.2; Mm01247058_m1) genes were obtained from TaqMan Pre‐Developed Assay Reagents (Applied Biosystems). A mouse Gapdh (GenBank accession no. NM_008084.2) TaqMan probe (4352339E, Applied Biosystems) was included as an endogenous control. The RT products (2 μl) were then subjected to real‐time PCR analysis in a final volume of 10 μl, with the use of TaqMan Fast Universal PCR Master Mix (Applied Biosystems) and with an ABI 7500 Thermocycler. Each sample was assayed in triplicate.

### 4.7 Immunofluorescence staining

Cells were washed in Tris‐buffered saline and fixed with 4% paraformaldehyde (Wako) overnight at 4°C. The fixed cells were permeabilized with 0.2% Triton X‐100 in Tris‐buffered saline for 30 min and washed in Tris‐buffered saline. In order to block nonspecific binding of antibodies, the cells were incubated at room temperature for 1 hr in 10% normal goat serum (Vector Laboratories) and 1% BSA (Sigma‐Aldrich) in Tris‐buffered saline. After incubation in blocking buffer, antibody staining was carried out as per standard procedures. Primary antibodies included goat polyclonal antibodies to Alb (1:200 dilution; A90‐134A; Bethyl Laboratories) and mouse polyclonal antibodies to Cyp1a2 (1:200 dilution; D‐51; Santa Cruz). Immune complexes were detected with Alexa Fluor 594‐conjugated antibodies to goat or mouse immunoglobulin G (each at 1:2,000 dilution; Molecular Probes). Antibodies were diluted in Tris‐buffered saline with 1% BSA. Cell nuclei were stained with Hoechst 33342 (5 μg/ml) in Tris‐buffered saline, and immunochemical staining was observed using an Olympus DP71 microscope.

### 4.8 Retroviral gene transfer

Mouse cDNAs for full‐length Foxa2, Hnf4a and Sall1 were obtained by digestion of OriGene plasmids (MR227662, MR227354 and MC203471) with NotI and BamHI, and the resulting fragments were cloned into the NotI and BamHI sites of retroviral plasmid pMEFs (kindly provided by H. Nobusue). The resulting vectors were designated pMEF‐Hnf4a‐IH, pMEF‐Foxa2‐IP and pMEF‐Sall1‐IB, and pMEFs‐GFP‐IB was used as a control. pMEF vectors were then transfected into Plat‐E packaging cells (Morita et al., 2000) using electroporation. The medium was later replaced once after 24 hr, and viral supernatants were collected and filtered using 0.45‐mm cellulose acetate filters (Millipore) 48 hr after transfection. Retroviral infection was carried out in a six‐well plate for 48 hr, and infected cells were subjected to selection in the presence of puromycin (5 μg/ml), hygromycin (50 μg/ml) or blasticidin (10 μg/ml).

### 4.9 Periodic Acid–Schiff's (PAS) staining

Cells were washed in Tris‐buffered saline and fixed in 1% periodic acid for 10 min. The fixed cells were then washed with distilled water and were exposed to Schiff's reagent (Wako) for 10 min. Then, cells were counterstained with hematoxylin (Wako) for 5 min and washed with distilled water. The dishes were imaged the next day using the DP71 Olympus microscope at a magnification of 100×.

### 4.10 Glycogen assay

Intracellular glycogen was assessed using the Glycogen Colorimetric/Fluorometric Assay Kit (BioVision). Samples were homogenized in dH_2_O, and they were immediately boiled in vented tubes at 100°C for 5 min to minimize glycogen degradation. Homogenates were then centrifuged at 16,000 *g* for 10 min, and the supernatant was assayed for glycogen according to the manufacturer's instructions. Total glycogen content was then normalized to total cellular protein concentration.

### 4.11 LDL uptake assay

LDL uptake by cells was assessed using fluorescence microscopy after incubation of the cells with 10 μg/ml LDL‐DyLight™ 550 (Cayman Chemical) for 4 hr at 37°C and Hoechst 33342 (5 μg/ml).

### 4.12 Measurements of Albumin

D‐Hep cells were cultured in the medium without phenol red. Culture supernatant was collected 24 hr after the medium change. According to the manufacturer's instructions, the amount of Alb in the supernatant was determined by the Mouse Albumin ELISA Kit (Shibayagi).

### 4.13 Cell viability tests

After hepatic induction of the FHS‐DFAT cells, the medium was replaced with fresh SHM medium, which contained the test compounds at the indicated final concentrations (troglitazone, acetaminophen and tamoxifen) (all from Wako). After exposure to the test compounds for 48 hr, the cell viability was examined by analyzing the intracellular ATP contents using the CellTiter‐Glo™ Luminescent Cell Viability Assay Kit (Promega), according to the manufacturer's instructions. The luminescent signals were measured with a Synergy 2 (BioTek). Control refers to incubations in the absence of test compounds and was considered as a 100% viability value.

### 4.14 Measurement of glutamic pyruvic transaminase (GPT)

The kits of GPT and GOT, provided by Transaminase C‐2 Test Wako (Wako), were used to measure the activity of GPT in 1.5 ml culture medium.

### 4.15 Determination of reduced glutathione (GSH)

GSSG/GSH Quantification Kits (Dojindo) were then used to measure the levels of intracellular GSH, according to the manufacturer's instructions.

### 4.16 Measurements of cytochrome P450 activity

Cytochrome P450 activity was measured after culture of DFAT cells, D‐Hep cells and adult mouse hepatocytes for 24 hr using a P450‐GloTM CYP1A2 Assay Kit (Promega), according to the manufacturer's instructions. The luminescent signals were measured using a Synergy 2 (BioTek).

### 4.17 Statistical analysis

Data are presented as mean ±* SD*. and were further analyzed with Tukey's honestly significant difference test or Student's *t* test for comparisons between two or among three or more groups, respectively. A *p*‐value <.05 was considered statistically significant.

## Supporting information

Figure S1
