# Supplementary material for: Generation of metabolically functional hepatocyte‐like cells from dedifferentiated fat cells by Foxa2, Hnf4a and Sall1 transduction
Source: Genes Cells. 2020 Nov 10;25(12):811–24. doi: 10.1111/gtc.12814 (PMC7894465; doi:10.1111/gtc.12814)
Supplement: Supplementary file 1 — Figure S1 [file GTC-25-811-s001.doc]

**Supplementary Figure S1**

**Supplementary Figure S1.** Flow chart of the chronological steps involved in the microarray analysis

Each box and the corresponding arrow display a major step in the gene expression analysis protocol using the microarray resources. The programs used for the analysis are indicated in the boxes.
